# Endothelial Dysfunction and Heart Failure with Preserved Ejection Fraction—An Updated Review of the Literature

**DOI:** 10.3390/life14010030

**Published:** 2023-12-24

**Authors:** Mariarosaria De Luca, Giulia Crisci, Giuseppe Armentaro, Sebastiano Cicco, Giovanni Talerico, Emanuele Bobbio, Lorena Lanzafame, Christopher G. Green, Abbie G. McLellan, Radek Debiec, Paolo Caferra, Roberto Scicali, Antonio Cannatà, Muhammad Zubair Israr, Liam M. Heaney, Andrea Salzano

**Affiliations:** 1 Department of Translational Medical Sciences, Federico II University, 80131 Naples, Italy; 2 Italian Clinical Outcome Research and Reporting Program (I-CORRP), 80131 Naples, Italy; 3 Department of Medical and Surgical Sciences, University Magna Græcia of Catanzaro, Campus Universitario di Germaneto, V.le Europa, 88100 Catanzaro, Italy; 4 Internal Medicine Unit “Guido Baccelli” and Arterial Hypertension Unit “Anna Maria Pirrelli”, Department of Precision and Regenerative Medicine and Jonic Area (DiMePReJ), University of Bari Aldo Moro, Azienda Ospedaliero-Universitaria Policlinico, 70124 Bari, Italy; 5 Internal Medicine, Policlinico Casilino, 00169 Rome, Italy; 6 Department of Cardiology, Sahlgrenska University Hospital, 413 45 Gothenburg, Sweden; 7 Institute of Medicine, The Sahlgrenska Academy, University of Gothenburg, Kuggen, 417 56 Gothenburg, Sweden; 8 Department of Clinical and Experimental Medicine, University of Catania, 95123 Catania, Italy; 9 School of Sport, Exercise and Health Sciences, Loughborough University, Loughborough LE11 3TU, UK; 10 Department of Cardiovascular Sciences, University of Leicester, Leicester (UK), IHR Leicester Biomedical Research Centre, Groby Road, Leicester LE3 9QP, UK; 11 Department of Pharmacy, University of Pisa, 56126 Pisa, Italy; 12 Department of Cardiology, King’s College Hospital NHS Foundation Trust, Denmark Hill, London SE5 9RS, UK; 13 Department of Cardiovascular Sciences, Faculty of Life Sciences & Medicine, King’s College, London SE1 8WA, UK; 14 Cardiac Unit, AORN A Cardarelli, 80131 Naples, Italy; 15 Cardiac Unit, University Hospital of Leicester, Glenfield Hospital, Leicester LE3 9QP, UK

**Keywords:** heart failure, endothelial function, endothelial dysfunction, diagnosis

## Abstract

Heart failure (HF) is a clinical syndrome consisting of typical symptoms and signs due to structural and/or functional abnormalities of the heart, resulting in elevated intracardiac pressures and/or inadequate cardiac output. The vascular system plays a crucial role in the development and progression of HF regardless of ejection fraction, with endothelial dysfunction (ED) as one of the principal features of HF. The main ED manifestations (i.e., impaired endothelium-dependent vasodilation, increased oxidative stress, chronic inflammation, leukocyte adhesion, and endothelial cell senescence) affect the systemic and pulmonary haemodynamic and the renal and coronary circulation. The present review is aimed to discuss the contribution of ED to HF pathophysiology—in particular, HF with preserved ejection fraction—ED role in HF patients, and the possible effects of pharmacological and non-pharmacological approaches. For this purpose, relevant data from a literature search (PubMed, Scopus, EMBASE, and Medline) were reviewed. As a result, ED, assessed via venous occlusion plethysmography or flow-mediated dilation, was shown to be independently associated with poor outcomes in HF patients (e.g., mortality, cardiovascular events, and hospitalization due to worsening HF). In addition, SGLT2 inhibitors, endothelin antagonists, endothelial nitric oxide synthase cofactors, antioxidants, and exercise training were shown to positively modulate ED in HF. Despite the need for future research to better clarify the role of the vascular endothelium in HF, ED represents an interesting and promising potential therapeutic target.

## 1. Introduction

Heart failure (HF) is a clinical syndrome caused by structural and/or functional abnormalities of the heart, resulting in elevated intracardiac pressures and/or inadequate cardiac output [1]; traditionally, HF is classified, based on the measurement of left ventricular ejection fraction (LVEF), in two main types: HF with reduced EF (HFrEF)—observed in patients with LVEF < 40%—and HF with preserved EF (HFpEF)—observed in patients with LVEF > 50%. Despite the vascular system playing a crucial role in the development and progression of HF regardless of the ejection fraction, in the last years, a growing body of evidence focused the interest of the scientific community on the association between endothelial function and HFpEF. Endothelial dysfunction (ED)—defined as the impairment of the complex balance in endothelial function—plays a critical role in the pathophysiology of HF [2]. The main manifestations of ED (i.e., impaired endothelium-dependent vasodilation, increased oxidative stress, chronic inflammation, leukocyte adhesion, and endothelial cell senescence) affect the systemic and pulmonary haemodynamics as well as the renal and coronary circulation [3]. The present review aims to highlight the contribution of ED to the pathophysiology of HF, its role in HF patients, and the potential effects of pharmacological and non-pharmacological approaches. For this purpose, relevant data from a literature search (PubMed, Scopus, EMBASE, and Medline) were reviewed.

## 2. Endothelial Dysfunction

### 2.1. Pathophysiology

**Figure 1:**
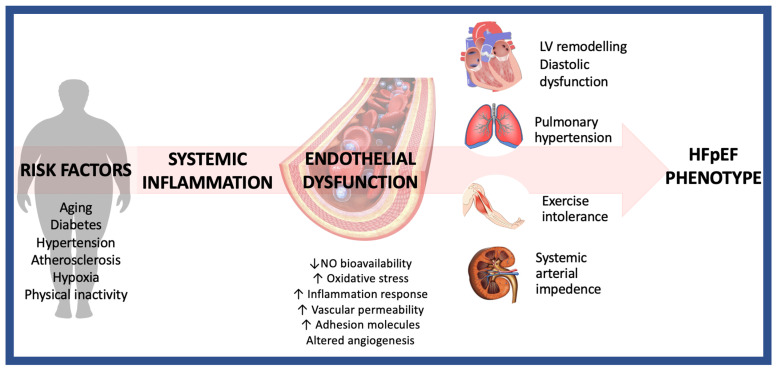
Role of endothelial dysfunction in determining HFpEF.

The healthy vascular endothelium, a dynamic organ, is a complex structural and functional barrier formed by a monolayer of cells [4]. It consists of vessels varying in size and functionality, persistently exposed to injury and repair processes [4]. Therefore, endothelial integrity is essential for several physiological processes in response to numerous chemical stimuli, such as neurotransmitters and hormones, or physical stimuli [5]. For instance, the endothelium participates in the haemostasis and thrombosis processes through the regulation of the clotting cascade, platelet aggregation, and fibrinolysis. Additionally, the endothelium produces inflammatory cytokines and regulates the adhesion of molecules [6]. Changes in endothelial function have also been correlated with the distinct activity of circulating endothelial progenitor cells (EPCs), which are considered potential cardiovascular indicators of organ damage such as left ventricular hypertrophy, heart failure, arterial stiffness, microalbuminuria, and altered glomerular filtration rate. EPCs are recruited from the bone marrow in response to vascular damage or tissue ischaemia. In the peripheral blood, EPCs contribute to reendothelialization and neovascularization, acting as positive regulators of haemostasis and vascular integrity [7]. A reduction in their number or function has been correlated with the development and progression of cardiovascular pathologies and major cardiovascular events [8]. Furthermore, both vasodilation and vasoconstriction are mediated by the endothelium subjected to the action of haemodynamic stimuli in response to changes in blood flow velocity or shear stress [6]. The compromission of this complex balance determines endothelial dysfunction (ED) (Figure 1).

### 2.2. Nitric Oxide

Endothelial effects on vascular tone are exerted through the production of different vasodilators and vasoconstrictors [9]. Nitric oxide (NO), the main vasoactive compound, is synthesized by endothelial cells and represents one of the main mediators of blood flow control [10]. NO is an inorganic, free-radical gas and influences haemostatic processes (e.g., adhesion and aggregation of platelets). It is essential for monocyte and leukocyte adhesion, counteracting the proliferation of smooth muscle cells, and assisting the oxidation of LDL cholesterol. A reduced NO production by endothelial cells, associated with an excess of reactive oxygen species, leads to impaired vasodilatory capacity and a proinflammatory and procoagulant systemic state [10]. This condition is considered a *primum movens* also in atherosclerotic disease and inner inflammatory changes. These mechanisms render atherosclerotic plaques unstable and prone to induce acute cardiovascular events [10,11,12,13,14]. On the other hand, when the endothelium is damaged, NO allows the release of endothelial progenitor cells from the bone marrow to repair the monolayer [15]. Various endogenous and exogenous factors contribute to stimulating the production and diffusion of NO through the vessel wall.

Nitric oxide (and L-citrulline as its by-product) is mainly synthesized from the N-guanine terminal of L-arginine and molecular oxygen in a reaction catalysed by nitric oxide synthase (NOS) enzymes [16]. In addition, NO can also be synthesized endogenously from circulating nitrate and nitrite [16]. Three isoforms of NOS, sharing half of their aminoacidic sequence identity, are well identified and classified as type 1 isoform nNOS (NOS1), type 2 isoform iNOS (NOS2) and type 3 isoform eNOS (NOS3) [17]. The expression of these isoforms is different in the various systems, and their synthesis can be regulated by calcium. In particular, calcium affects nNOS activity and is necessary for neuronal signalling through the release of norepinephrine at nerve endings by a Ca^2+^-dependent mechanism. The eNOS isoform is constitutively produced in different cell types, such as endocardial cells and endothelial cells. Finkel et al. demonstrated that the inhibitory effects of the pro-inflammatory cytokines tumour necrosis factor-alpha (TNF-α), interleukin-2 (IL-2), and interleukin-6 (IL-6) on the contractility of isolated papillary muscles in hamsters were associated with the repression of myocardial NO production through the activity of eNOS [18]. Meanwhile, iNOS is an inducible Ca^2+^-independent isoform, stimulated by cytokines or bacterial endotoxins [16]. During the activation of the inflammatory and innate immune system, iNOS plays a key role in helping to defend against damage from pathogens. However, when overexpressed or dysregulated, iNOS activity can be dangerous, as it catalyses the synthesis of NO with cytotoxic and negative inotropic effects [19].

### 2.3. Clinical Assessment of the Endothelial Function

The clinical assessment of the endothelial function is performed by either invasive procedures, such as intracoronary assessment, or non-invasive procedures, such as brachial artery flow-mediated dilation (FMD) measurement (Table 1). The first examination of ED was based on the use of acetylcholine in the coronary circulation [20]. When the drug was infused in atherosclerotic coronary arteries, the vascular response to endothelium-dependent stimuli was evaluated with quantitative angiography and intracoronary doppler assessment [21]. The parameters evaluated were coronary diameter and blood flow. However, these techniques are invasive and expensive, and as a result, their use is limited almost exclusively to the research field.

Since these early studies, many researchers have assessed ED using less invasive methods to identify alterations in the endothelia function. Among these non-invasive methods, venous occlusion plethysmography (VOP) allows the non-invasive measurement of the blood flow in the forearm for peripheral endothelial assessment [22]. This technique permits human in vivo vascular physiology assessment; specifically, after a local anaesthesia (not mandatory), the local brachial artery infusion of an endothelium-dependent agonist (i.e., acetylcholine) is followed by arresting the venous outflow using a cuff placed around the arm; usually, the cuff is inflated at a sub-diastolic pressure of 40 mmHg every 5–10 s [23], which is enough to obstruct the venous outflow while preserving the arterial inflow. During the test, the circulation of the hand is excluded through the use of a smaller cuff placed around the wrist, inflated at a suprasystolic pressure (220 mmHg for normotensive subjects). By this method, changes in the forearm circumference (directly proportional to changes in blood flow when normalised to the forearm volume) are measured by using a voltage-dependent strain gauge and recorded on a plethysmograph. This method is accurate and reproducible. Specifically, two studies demonstrated the predictive role of cardiovascular events using this method to evaluate ED [24,25].

Among the non-invasive methods, flow-mediated dilation (FMD) assessment is the most reproducible technique to evaluate changes in brachial artery diameter [3]. This technique was first used in 1992 and exploits a physiological mechanism regulating vascular tone and homeostasis in the peripheral circulation. To perform this the test, a cuff is positioned below the antecubital fossa and is inflated to suprasystolic pressure for 5 min; during this period of obstruction, ischaemia in distal tissues causes the distal vessels to dilate, lowering vascular resistance [26]. After the arm cuff is released, there is a reduction in downstream resistance, which increases the blood flow to the arm. As a result, the endothelium responds to the subsequent increase in shear stress by releasing vasodilators, including nitric oxide, which cause dilation in a healthy artery; on the other hand, if ED is present, dilation is reduced or absent. After arm cuff release, the flow is measured with ultrasound, and the baseline and peak diameters are used to calculate FMD as absolute and percent changes in artery diameter (i.e., mm and %). Notably, despite brachial FMD being an operator-influenced method, it correlates well with invasive measurements, allowing the measure of arterial dilation in response to shear stress forces [27,28].

Another simple, reproducible, and cost-effective method to assess ED is the ankle–brachial index (ABI). This method calculates the ratio of systolic pressure measured at the ankle to that measured at the brachial artery. The ABI indirectly indicates ED, mainly related to lower limb atherosclerosis. It has the advantage of being cost-effective and easily and quickly available also in nonspecialized centres [29].

Peripheral arterial tonometry (PAT) consents a non-invasive measure of ED. It uses finger plethysmography to evaluate pulse wave amplitude (PWA) [30]. By applying a sub-diastolic pressure at the middle and distal phalanges of a finger, a computer algorithm calculates the reactive hyperaemia-PAT index (RHI) as the ratio of the PWA signal after cuff release to that at baseline. These measurements are compared with those obtained for the contralateral arm. Several investigators have shown that the RHI is a predictor of cardiovascular events [31,32].

The analysis of the endothelium permits the recognition of subclinical alterations in vascular function and has as a prognostic value for future cardiovascular (CV) events [33]. Indeed, several studies assessed its value as a predictor of cardiovascular risk and as a predictor of poor outcomes in patients with established CV disease. For example, the assessment of the endothelial vasodilator function was evaluated by Schachinger et al., who demonstrated a high incidence of cardiovascular events in patients with alterations in coronary vasomotion and mild coronary disease [34].

### 2.4. Clinical Assessment of Coronary Microvascular Dysfunction

In recent years, the role of coronary microvascular dysfunction (CMD) in CV diseases and HF pathophysiology has been demonstrated (Table 1). CMD is defined as an abnormal response to physiological or pharmacological stress of the myocardial microcirculation, leading to inadequate vasodilation [35]. A range of invasive and non-invasive tests are required to evaluate the presence of CMD.

Thrombolysis in myocardial infarction (TIMI) frame count and TIMI myocardial perfusion grades measured during invasive coronary angiography are surrogate measures of CMD; however, they do not provide information about the underlying mechanisms (impaired dilation or microvascular spasm) [36]. To obtain a comprehensive assessment of the coronary microcirculation, investigation of the vasodilator and vasoconstrictor microvascular responses is necessary. Other modalities (e.g., use of intracoronary temperature–pressure wire and intracoronary Doppler flow–pressure wire) allow for the evaluation of coronary flow reserve (CFR), hyperaemic microvascular resistance (HMR), and fractional flow reserve (FFR) [37].

Non-invasive techniques can be used to identify CMD in subjects in which the presence of obstructive coronary artery disease has been excluded using computer tomography or invasive coronary angiography. Among these, positron emission tomography (PET) is the gold standard. PET evaluates the myocardial perfusion reserve (MPR) in all coronary territories by quantification of the myocardial blood flow (MBF) at rest and during pharmacologically induced maximal hyperaemia. Transthoracic Doppler echocardiography allows the evaluation of the maximal diastolic flow in the coronary arteries during rest and stress, known as coronary flow velocity ratio (CFVR) [38]. Finally, myocardial computed tomography scanning and CMR allow a semi-quantitative assessment of MBF and MPR [36].

## 3. Endothelial Dysfunction in Chronic Heart Failure

Endothelial dysfunction represents a subclinical alteration commonly found in cardiovascular diseases, leading to several clinical expressions [3]. In fact, the impairment of the endothelial function has a crucial role in the pathogenesis and progression of atherosclerosis, promoting the thickening of the vessel wall and plaque formation. Furthermore, it is a pathophysiological movens of different pathological conditions such as arterial hypertension, diabetes mellitus, hypercholesterolaemia, and heart failure (HF) [3,39]. Oxidative stress is augmented in these pathological conditions, leading to ED. Thus, endothelial dysfunction in turn contributes to abnormal cardiac and vascular phenotypes.

A reduction in NO bioavailability is one of the initial steps in the atherosclerotic process, with evidence showing it as a predictive factor of cardiovascular events. Therefore, the analysis of the endothelial function allows for the evaluation of cardiovascular risk in patients lacking angiographical coronary alterations [33]. In HF patients, not only are these mechanisms impaired, but also dysfunction varies in different circulatory districts. Furthermore, the assessment of coronary and peripheral endothelial dysfunction is variable in different organ systems. In this regard, Elkayam et al. demonstrated that the response to vasodilators in HF patients is preserved in the renal district but impaired in the coronary and pulmonary circulation [40]. Traditionally, systemic vasoconstriction has been considered a hallmark of advanced chronic congestive HF. Recent findings suggested that endothelial dysfunction has a crucial role in the pathophysiology of HF [3,41]. Specifically, it is one of the early mechanisms that determine a reduction in organ perfusion, intolerance to physical efforts, and the advancement of HF. The impairment of endothelial cell function is determined by numerous alterations, e.g., the activation of the sympathetic nervous system, renin–angiotensin system, and pituitary–vasopressin axis, as well as an increase in a systemic pro-inflammatory state associated with the release of cytokines, such as TNF-α, IL-1, and IL-6. In particular, NO production is unbalanced in HF patients, with an increase in oxidative stress and a decrease in bioavailable NO. The alteration of the redox state is a mechanism responsible for the decline of cardiac performance, initiated through direct damage caused by myocardial ischaemia [42]. Therefore, NO reduction in HF occurs through various mechanisms: a reduction in the synthesis of NO from eNOS activity, increased NO degradation, and increased expression of endothelin 1 (ET-1). In particular, several neurohumoral factors widely produced in chronic HF stimulate cardiac iNOS expression and determine cardiac alterations with consequent myocardial dysfunction. Furthermore, proinflammatory cytokines down-regulate eNOS expression and their levels are correlated with a progressive impairment of the endothelial function; in addition, the overexpression of TNF-α may aggravate skeletal muscle atrophy and is associated with impairment of exercise capacity, a common feature in HF patients [43,44,45]. Finally, ET-1 is a peptide produced by endothelial cells that exerts its action on smooth muscle cells, with receptors localized on vascular walls. In fact, via ET-A and ET-B receptor binding on vascular smooth muscle cells, ET-1 promotes vasoconstriction. Conversely, the binding of ET-B on endothelial cells determines vasorelaxation. In HF, the augmentation of ET-1 expression causes the growth of smooth muscle cells and matrix production, leading to increased vascular resistance and consequent remodelling [46]. Furthermore, high levels of ET-1 are associated with worse physical performance and higher mortality in heart failure patients [36]. Several findings suggested positive effects of exercise on heart failure. In fact, recent guidelines recommend cardiac rehabilitation to help manage heart failure; notably, cardiac rehabilitation leads to an improvement of the endothelial function, assessed by FMD [47]. Specifically, exercise training was shown to improve endothelium-dependent vasodilation mediated by NO. Physical activity increased shear stress, leading to augmented NO production, upregulation of eNOS expression, and stimulation of antioxidative enzymes [48,49]. Furthermore, exercise in patients with HF reduced cardiac apoptosis and proinflammatory markers and improved muscle performance.

## 4. Evidence of Endothelial Dysfunction in Heart Failure with Preserved Ejection Fraction (HFpEF)

Despite the first studies documenting scientific interest in the relationship between HFpEF and endothelial function around two decades ago, to date the investigation of endothelial dysfunction in HFpEF patients is still limited (Table 2). The first investigation was performed by Hundley and colleagues in 2007, who performed a cardiovascular magnetic resonance (CMR) assessment following FMD of the superficial femoral artery in a cohort of 30 elderly participants (11 healthy subjects, 9 patients with HFpEF, and 10 patients with HFrEF) [50]. FMD was 3.8 ± 1.3% (0.85 ± 0.22 mm^2^) in patients with HFrEF, 12.1 ± 3.6% (3.1 ± 1.2 mm^2^) in patients with HFpEF, and 13.7 ± 5.9% (3.9 ± 1.7 mm^2^) in healthy controls. After multivariate analysis with intergroup differences in patient characteristics, FMD between HFpEF and HFrEF patients was statistically different, whilst FMD between HFpEF patients and controls was not. Notably, both HF groups showed similar severe reductions in exercise V̇O_2_ [50]. A few years later, the same research group extended their results by examining FMD in a different arterial bed (brachial), showing no differences between patients and healthy controls, despite impaired cardiopulmonary performance in the former [51]. Taken together, even if with the limitations of small sample sizes as well as the presence of conditions that would influence FMD in the HFpEF group (absence of advanced arteriosclerosis, diabetes, or hypercholesterolemia), the final findings supported the hypothesis that an abnormal FMD is not a significant contributor to severe exercise intolerance in HFpEF patients [51]. However, this theory has been questioned by more recent studies. In a study by Borlaug et al., subjects with HFpEF (*n* = 21), hypertension without heart failure (*n* = 19), or no cardiovascular disease (*n* = 10) were studied at rest and during exercise. For the first time, endothelial function was assessed in HFpEF subjects and compared to that in healthy controls at the microvasculature level. Exercise PAT amplitude with reactive hyperaemia was diminished in HFpEF patients and hypertensive subjects when compared with control subjects, suggesting endothelial dysfunction. Specifically, the prevalence of endothelial dysfunction was 42% in HFpEF subjects (*p* < 0.05 vs. control), 28% in hypertensive subjects (*p* = 0.056 vs. control), and 0% in the controls. The authors suggested that this deficit could be partially linked to atherosclerosis through reactive hyperaemia index (RHI) analysis, which showed impairments in HFpEF subjects after adjusting for coronary disease, with mean RHI values similar in HFpEF patients with or without coronary disease. In brief, in contrast to previous studies, endothelial dysfunction was associated with reduced exercise tolerance and greater symptoms such as dyspnoea and fatigue, suggesting a contributary role to exercise intolerance in HFpEF [52]. The microvasculature findings of this study were confirmed by subsequent data from independent groups, which showed altered values of forearm cutaneous blood flow [53] and of PAT [54] in HFpEF patients vs. controls [54].

Other investigations comprehensively evaluated the vascular function at the macrovascular level via the FMD of conduit arteries and at the microvascular level via post-occlusive reactive hyperaemia. A study comparing 24 HFpEF patients to 24 healthy controls showed a reduction in brachial artery FMD, assessed by the peak percentage (HFpEF patients: 3.06 ± 0.68%, controls: 5.06 ± 0.53, *p* = 0.03), and an absolute change in brachial artery diameter (HFpEF patients: 0.13 ± 0.03 mm, controls: 0.23 ± 0.12 mm, *p* = 0.02) in HFpEF patients. The impairment in FMD vanished when FMD was normalized considering the shear stimulus. In contrast, the RHI was impaired in patients with HFpEF during the initial 50 s following cuff release compared with the controls (*p* = 0.03). According to the authors, in combination, these findings suggest that the vascular damage in HFpEF patients is primarily driven by a dysfunctional microcirculation [55]. Similarly, a recent report evaluated the endothelial function of both conduit arteries and microvasculature in HFrEF and HFpEF patients via FMD and RHI measurements. No statistically significant differences were observed between the two groups for both parameters. In the subgroup of patients with ischaemic heart disease, the degree of FMD was comparable, independently of the HF phenotypes, but the RHI was lower in HFrEF patients compared to HFpEF patients (*p* = 0.014). In contrast, in non-ischaemic patients, the degree of FMD was lower in HFpEF patients compared to HFrEF patients (*p* = 0.009), whereas the RHI was similar in the two groups. These results suggest that the clinical significance of FMD and RHI measurements in patients with HF may diverge depending on the concomitant presence of coronary disease, rather than on the HF phenotype based on EF [56].

In addition to vascular dysfunction, abnormalities of the vascular structure may contribute to the pathogenesis and maintenance of HFpEF. Indeed, a recent study by Kishimoto et al. demonstrated, for the first time, that both endothelial dysfunction and abnormal vascular structure in the same artery are associated with HFpEF [57]. The authors measured FMD and nitroglycerine-induced vasodilation to assess vascular function and intima–media thickness (IMT) and examine the vascular structure of the brachial artery in 41 HFpEF patients and 165 controls. FMD was lower in patients with HFpEF than in controls (2.9 ± 2.1% versus 4.6 ± 2.7%, *p* = 0.0002). Similarly, nitroglycerine-induced vasodilation was lower in patients with HFpEF than in controls (9.3 ± 4.1% versus 12.9 ± 4.9%, *p* < 0.0001). Brachial artery IMT was higher in patients with HFpEF than in patients without HF (0.35 ± 0.06 mm versus 0.31 ± 0.07 mm, *p* = 0.0002). In brief, this study demonstrated advanced vascular failure in patients with HFpEF, involving both impaired endothelial function and vascular structure [57].

Recent studies reported a high prevalence of pulmonary hypertension (PH) in HFpEF patients, determining a worse prognosis [58]. Although the mechanisms underlying the high prevalence of PH are unknown, Ferrero et al. speculated that patients with HFpEF suffer from endothelial dysfunction that affects both the pulmonary and the peripheral vasculature and that peripheral endothelial dysfunction assessment could reflect pulmonary endothelial dysfunction and, therefore, be related to the presence of PH. Thus, they performed an observational study aimed to analyse the association between peripheral endothelial dysfunction and pulmonary hypertension in patients with HFpEF. The results showed that, following adjustments for age, sex, and nitrate use, the HFpEF patients had impaired peripheral endothelial function when compared with hypertensive control patients, as shown by their lower FMD [1.95% (−0.81 to 4.92) versus 5.02% (3.90 to 10.12), *p* = 0.002]. Moreover, HFpEF patients with systolic pulmonary artery pressure >35 mmHg on echocardiogram also underwent a right heart catheterization that highlighted an inverse correlation between FMD and pulmonary vascular resistance in patients with HFpEF and pulmonary hypertension. The authors speculated that the worse prognosis for patients with HFpEF and peripheral dysfunction could be related to the presence of pulmonary hypertension [59].

**Table 2:** Results of trials assessing peripheric endothelial dysfunction in patients with heart failure with preserved ejection fraction.

First Author, Year of Publication Ref.	Sample Size and Population	Methodology	Major Findings
Hundley WG, 2007 [50]	9 HFpEF 10 HFrEF 11 controls	CMR assessment of FMD of the superficial femoral artery	HFpEF: 12.1 ± 3.6% HFrEF patients 3.8 ± 1.3% Controls: 13.7 ± 5.9% *p*: ns (HFpEF vs. controls) *p*: <0.001 (HFpEF vs. HFrEF)
Borlaug BA, 2010 [52]	21 HFpEF 19 Hypertensives with no HF 10 Controls	PAT (Log RHI)	HFpEF: 0.85 ± 0.42 Hypertensives: 0.92 ± 0.38 Controls: 1.33 ± 0.34 *p*: <0.05 (HFpEF and hypertensives vs. controls)
Haykowsky MJ, 2013 [51]	66 Older HFpEF 31 Older controls 16 Young controls	Brachial artery FMD	HFpEF: 3.64 ± 0.28% Older controls: 4.00 ± 0.38% Young controls: 6.13 ± 0.53% *p*: ns (HFpEF vs. older controls) *p*: 0.0001 (HFpEF vs. young controls) *p*: 0.0002 (older vs. young controls)
Farrero M, 2014 [59]	28 HFpEF 42 Hypertensive controls	Brachial artery FMD	Patients: 1.95 (−0.81–4.92) % Controls: 5.02 (3.90–10.12) % *p* = 0.002
Maréchaux S, 2016 [53]	45 HFpEF 45 Hypertensive controls	Brachial artery FMD	Patients: 3.6 (0.4–7.4) % Controls: 7.2 (3.2–12.7) % *p* = 0.001
		Forearm cutaneous blood flow at rest	Patients: 33 (14–61) PU Controls: 39 (17–62) PU *p* = 0.68
		Forearm cutaneous blood flow after arterial occlusion	Patients: 135 (104–206) PU Controls: 177 (139–216) PU *p* = 0.03
Lee JF, 2016 [55]	24 HFpEF 24 Controls	Brachial artery FMD (peak percentage)	Patients 3.06 ± 0.68% Controls 5.06 ± 0.53% *p* = 0.03 No difference when normalized for the shear stimulus
		Brachial artery FMD (absolute change in brachial artery diameter)	Patients 0.13 ± 0.03 mm Controls 0.23 ± 0.12 mm *p* = 0.02
		RH (AUC)	Patients:454 ± 35 mL/min Controls: 659 ± 63 mL/min *p* < 0.01
Kishimoto S, 2017 [57]	41 HFpEF 165 control subjects with cardiovascular risk factors	Brachial artery FMD	Patients 2.9 ± 2.1% Controls 4.6± 2.7% *p* = 0.0002
		Nitroglycerine-induced vasodilation	Patients 9.3 ± 4.1% Controls 12.9 ± 4.9% *p* < 0.0001
		Brachial artery intima media thickness	Patients 0.35 ± 0.06 mm Controls 0 0.31 ± 0.07 mm *p* = 0.0002
Gevaert AB, 2019 [54]	26 HFpEF 24 Healthy controls, matched for age and sex	PAT (Log RHI)	Patients 1.8 (2.0–3.3) Controls 1.9 (1.6–2.9) *p* = 0.036
Waku R, 2020 [56]	42 HFpEF 46 HFrEF	Brachial artery FMD	HFpEF 4.14 ± 2.24% HFrEF 4.98 ± 2.38% *p* = 0.093
		PAT (Log RHI)	HFpEF 1.81 (1.60–2.25) HFrEF 1.60 (1.42–2.10) *p* = 0.059

CMR: cardiovascular magnetic resonance; FMD: flow-mediated dilatation; HFpEF: heart failure with preserved ejection fraction; HFrEF: heart failure with reduced ejection fraction; PAT: peripheral arterial tonometry; PU: perfusion unit; RH: reactive hyperaemia.

A recent meta-analysis including a total of seven studies that evaluated the impact of HFpEF on FMD and/or nitrate-mediated dilation (NMD) confirmed impaired endothelial function in HFpEF patients [51]. Moreover, regression models showed that differences in echocardiographic findings (i.e., E/A ratio, E/e’ ratio, and left atrial diameter) were positively associated with higher differences in FMD values between cases and controls [E/A ratio (Z-score: −2.002; *p* = 0.045), E/e’ ratio (Z-score: −2.181; *p* = 0.029) and left atrial diameter (Z-score: −1.951; *p* = 0.050)]. In contrast, an increased use of calcium channel blockers was associated with a lower effect size [60]. 

## 5. Coronary Microvascular Dysfunction in Heart Failure with Preserved Ejection Fraction

Endothelial dysfunction and coronary myocardial dysfunction (CMD) play a fundamental direct role in the development of cardiac remodelling and diastolic dysfunction in HFpEF patients [61].

In a small prospective observational study, Dryer et al. compared 30 HFpEF patients to 14 controls. All subjects underwent cardiac catheterization, whilst CFR and the index of microvascular resistance (IMR) measurements after adenosine administration were determined. The thresholds of CFR ≤ 2.0 and IMR ≥ 23 were used [62]. HFpEF patients had lower CFR (2.55 ± 1.60 vs. 3.84 ± 1.89, *p* = 0.024) and higher IMR (26.7 ± 10.3 vs. 19.7 ± 9.7 units, *p* = 0.037) than control subjects. Moreover, one-third of the HFpEF cohort exhibited overt CMD [63]. Yang et al. enrolled 162 consecutive HFpEF patients referred for invasive coronary haemodynamic assessment. Endothelial function was quantified by the increase in coronary blood flow in response to the intracoronary infusion of acetylcholine using a Doppler flow wire with quantitative angiography. Endothelium-independent CMD was defined as CFR ≤ 2.5 [64], and endothelium-dependent CMD was defined as an increase in coronary blood flow (CBF) ≤10% in response to acetylcholine [65]. Overall, CMD was present in 72% of the patients (29% with isolated endothelium-dependent CMD, 33% with endothelium-independent CMD, and 10% with combined CMD forms). Over a median follow-up of 12.5 years, the patients with endothelium-dependent CMD revealed a trend of increased mortality compared with those with preserved endothelial function (adjusted hazard ratio, 2.81, 95% CI, 0.94–8.34, *p* = 0.06). Overall, mortality was significantly higher for the patients with HFpEF and endothelium-independent CMD vs. the patients with CFR  >  2.5 (adjusted hazard ratio, 3.56, 95% CI, 1.14–11.12, *p* = 0.03) [66]. A large prospective, multicentre, cohort study enrolled 106 consecutive patients hospitalized with HFpEF, revealing endothelium-independent CMD (i.e., coronary flow reserve <2.0 and/or index of microvascular resistance ≥25) in 66% of the participants and endothelium-dependent CMD (i.e., abnormal coronary vasoreactivity) in 24% of the participants. Overall, 85% of the subjects showed evidence of CMD, and 81% of those without obstructive epicardial CAD had CMD. In this study, the presence of CMD showed no significant association with prognosis [67]. Furthermore, CMD has also been shown to be a hallmark of all pathologies presenting with a diagnosis of myocardial infarction without obstructive coronary artery disease (MINOCA), as assessed based on the coronary microvascular function in all three coronary territories by means of the angiography-based index of microvascular resistance (aIMR) [68]; intriguingly, CMD is also linked to MINOCA through Killip classification [69]. In summary, invasive studies showed that the prevalence of CMD in HFpEF patients ranges between 70% and 85% depending on the diagnostic cut-off used for CFR and IMR, and these data were confirmed in studies performing non-invasive assessments of the coronary vascular function. Recently, the multinational PROMIS-HFpEF (PRevalence Of MIcrovascular dySfunction in Heart Failure with Preserved Ejection Fraction) study showed that among 202 patients with HFpEF, 151 had CMD (defined, using adenosine stress transthoracic Doppler echocardiography as CFR < 2.5), and a prespecified exploratory analysis found that CMD was also independently associated with cardiovascular and HF-specific events at a 1-year follow-up [70,71].

## 6. Prognostic Implications

Even though the prognostic role of endothelial dysfunction in HFpEF patients remains to be fully clarified, several potential pathophysiological mechanisms may contribute to disease progression and consequent poor outcomes in HFpEF patients, such as pathological ergoreflex activation—related to NO bioavailability—and NO-related myocardial perfusion abnormalities. A non-randomized retrospective study enrolling 159 HFpEF patients identified endothelial dysfunction, measured by PAT, as an independent predictor of HF-related events (HF-related death and re-hospitalization due to congestive heart failure at 300 days). This finding was confirmed after multivariate analysis considering other established risk factors for adverse events in HFpEF patients. The authors identified a logRHI cut-off of 0.49 via ROC analysis as the optimal cut-off point of the logarithmic reactive hyperaemia index for the prediction of prognosis in HFpEF patients [72]. In the same year, Akiyama et al. published a prospective cohort study evaluating peripheral endothelial function by RH-PAT and cardiovascular outcomes in 320 HF patients with EF > 50%. The mean follow-up period was 20 months, during which 59 cardiovascular events occurred. The frequency of the cardiovascular events was higher in the low-RHI group (below the median, cut-off value: 0.49) compared with the high-RHI group (above the median), *p* < 0.001. Kaplan–Meier analysis confirmed a significantly higher probability of cardiovascular events in low-RHI patients compared with high-RHI patients [73]. The above-mentioned investigation by Yang et al. confirmed a trend of worse outcomes in HFpEF patients with endothelium-dependent CMD [66].

## 7. Future Therapeutical Approaches

A timely diagnosis of HFpEF remains a challenge. Moreover, to date, no therapy has demonstrated improvements in patient prognosis. Endothelial dysfunction represents an attractive therapeutic target in HFpEF patients due to its systemic involvement and reversibility in the early stages of the disease. As per HFrEF [39,74,75], ED in HFpEF can be improved by the correction of comorbidities, NO bioavailability, and oxidative status [76]. Thus, it has been hypothesized that therapies targeting generalized vascular dysfunction, such as anti-inflammatory drugs, antioxidants, anti-vascular permeability drugs (glycocalyx, pericyte, and cell–cell junction stabilizers), and angiogenic therapy might ameliorate HFpEF patients’ general health [77]. The results, however, are rather limited. A small study assessed the effect of sildenafil on 48 HFpEF patients, administered over 24 weeks, with no significant improvement of the reactive hyperaemic change in digital blood flow [78].

The recent results from the DELIVER and EMPEROR-Preserved trials with the administration of sodium–glucose cotransporter 2 (SGLT2) inhibitors in HFpEF patients represent a significant step forward in the treatment of HFpEF [79,80]. The mechanisms by which gliflozins improve the disease prognosis in these patients are not yet fully understood, but a role in endothelial function regulation cannot be excluded [79,80]. In this context, Kolijn et al. carried out a pre-clinical in vitro study using human myocardium from patients with HFpEF and murine models. They showed that empagliflozin reduced inflammatory and oxidative stress, besides improving the NO–sGC–cGMP–cascade and PKGIα activity [81]. Numerous pathophysiological mechanisms could promote an improvement of vascular parameters in patients treated with SGLT2is, such as a reduction in waist circumference, body weight, and blood pressure [82]. Moreover, SGLT2i could exert *per se* vascular effects, as suggested by the limited available evidence [67,68]. In the DEFENCE study, 16 weeks of dapagliflozin therapy improved the endothelial function, as assessed by FMD, in 80 diabetic patients [83]. Similarly, diabetic patients on acute treatment with dapagliflozin reported significantly improved systemic endothelial function, arterial stiffness, and renal resistive index, independently of their blood pressure [84]. However, the patients enrolled in these two studies had no HFpEF. Moreover, experimental and clinical studies demonstrated that SGLT2is exert protective effects on renal physiology by improving renal endothelial dysfunction and inflammation, both in diabetic and in non-diabetic patients [85,86,87,88].

Exercise intolerance represents a typical feature of HFpEF, and exercise training programmes have been shown to ameliorate cardiopulmonary performances, as shown by improvements in V̇O_2_ peak, ventilatory threshold, and 6 min walking test distance [89,90]. A single mechanism underlying exercise intolerance in patients with HFpEF cannot be identified, but endothelial dysfunction was identified as a contributor to its pathophysiology. Kitman et al. investigated the effects of regular exercise on peripheral vascular function. This study randomized 63 elderly patients with HFpEF to 16 weeks of exercise training (walking, arm, and leg ergometry) or attention control. The observed findings included an improvement in peak V̇O_2_ in the exercise training group, without a change in brachial artery FMD and carotid arterial distensibility, which were the primary outcomes of the study [91]. Similarly, in a study by Angadi et al., 4 weeks of high-intensity interval training improved peak V̇O₂ and left ventricular diastolic dysfunction grade, but FMD was unchanged [92]. These studies suggest that exercise training improves exercise tolerance in patients with HFpEF, but this improvement is likely independent of changes in flow-mediated dilation. In contrast, functional electrical stimulation of lower limbs improved exercise capacity and FMD in patients with HFpEF [93].

## 8. Conclusions

There is an intimate link between HFpEF and endothelial function, involving pathological processes and multiple systems. Both coronary and peripheral endothelial dysfunction have been associated with HFpEF and its adverse evolution, suggesting a role for systemic microvascular disease in HFpEF development. Despite this, the prognostic role of ED has not yet been fully elucidated. Several potential pathophysiological mechanisms may contribute to HFpEF progression. Recent research revealed an association between SGLT2 inhibition and improved endothelial function. Determining the role of endothelial function in HFpEF and exercise limitation as well as targeting physiologic abnormalities could be useful to establish tailored therapies based on the dominant HFpEF syndrome phenotype.

## Figures and Tables

**Table 1:** Main techniques for the measurement of endothelial function.

Technique	Site	PROS	CONS
Venous occlusion plethysmography	Venous system of limbs	Non-invasive Accuracy	Strict protocol to follow
Flow-mediated dilation	Forearm	Non-invasive High reproducibility Correlation with invasive measurements	Operator skill required Strict protocol to follow
Ankle–brachial index	Lower limb	Non-invasive Easily and quickly obtainable Cost-effective	Indirect measure of ED
Peripheral arterial tonometry	Microvasculature of fingertips	Non-invasive Operator independent Easy to use	Strict protocol to follow Costly probes
Thrombolysis in myocardial infarction (TIMI) frame count and TIMI myocardial perfusion grades	Coronary Microvacular disease	Accuracy Relatively easy	Invasive No information about ED underlying mechanisms
Coronary flow reserve	Coronary Microvacular disease	Feasible Safe Reproducible	Invasive Requires hyperaemia Operator-dependent

## Data Availability

Not applicable.
